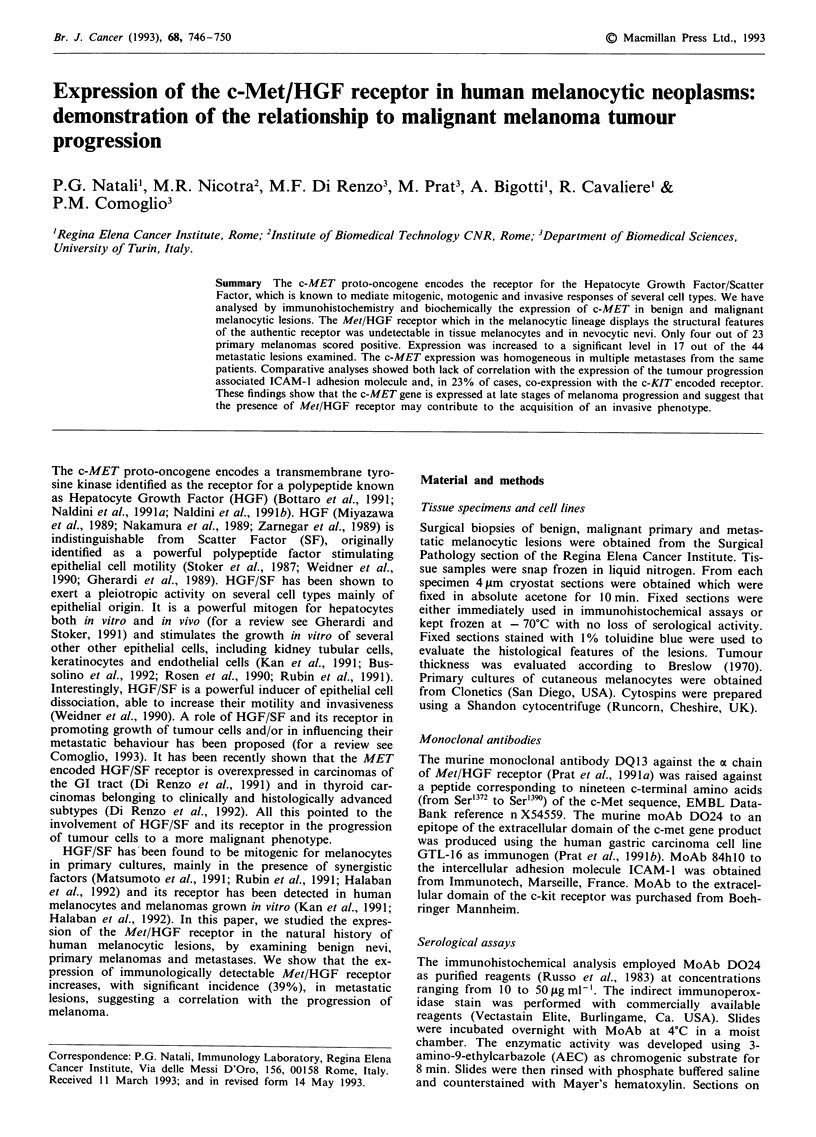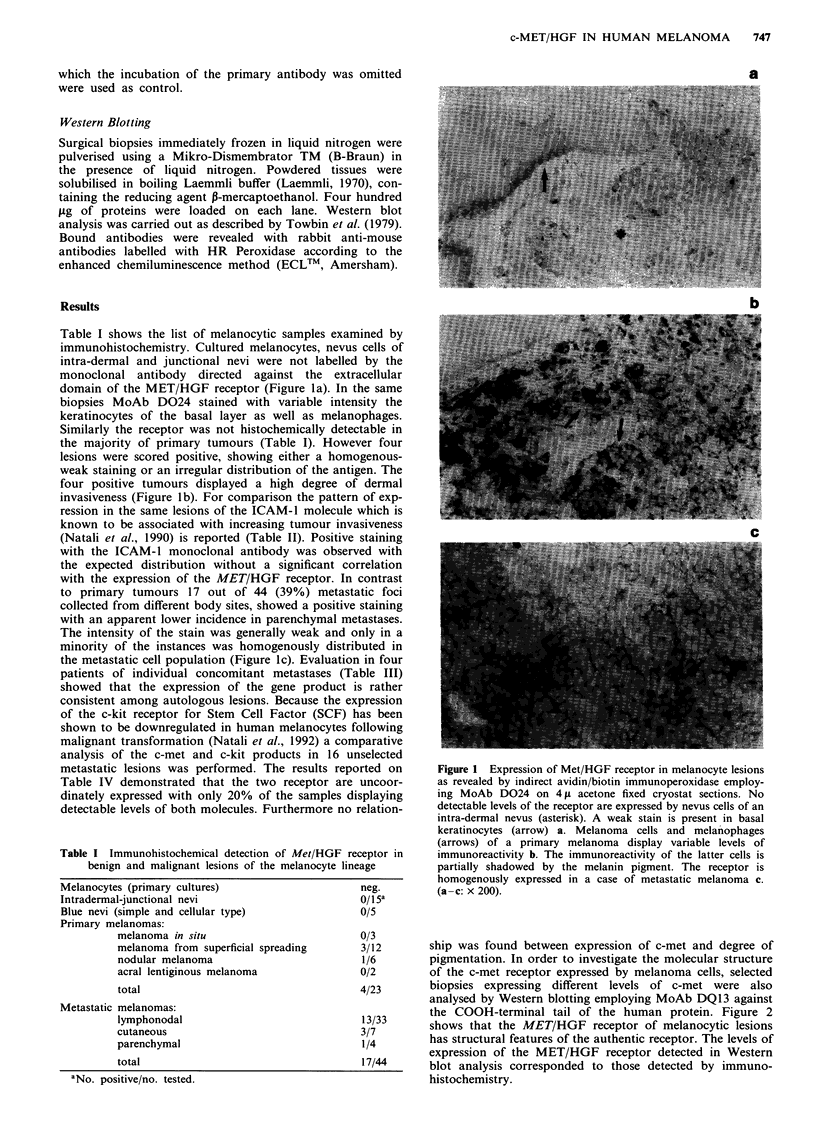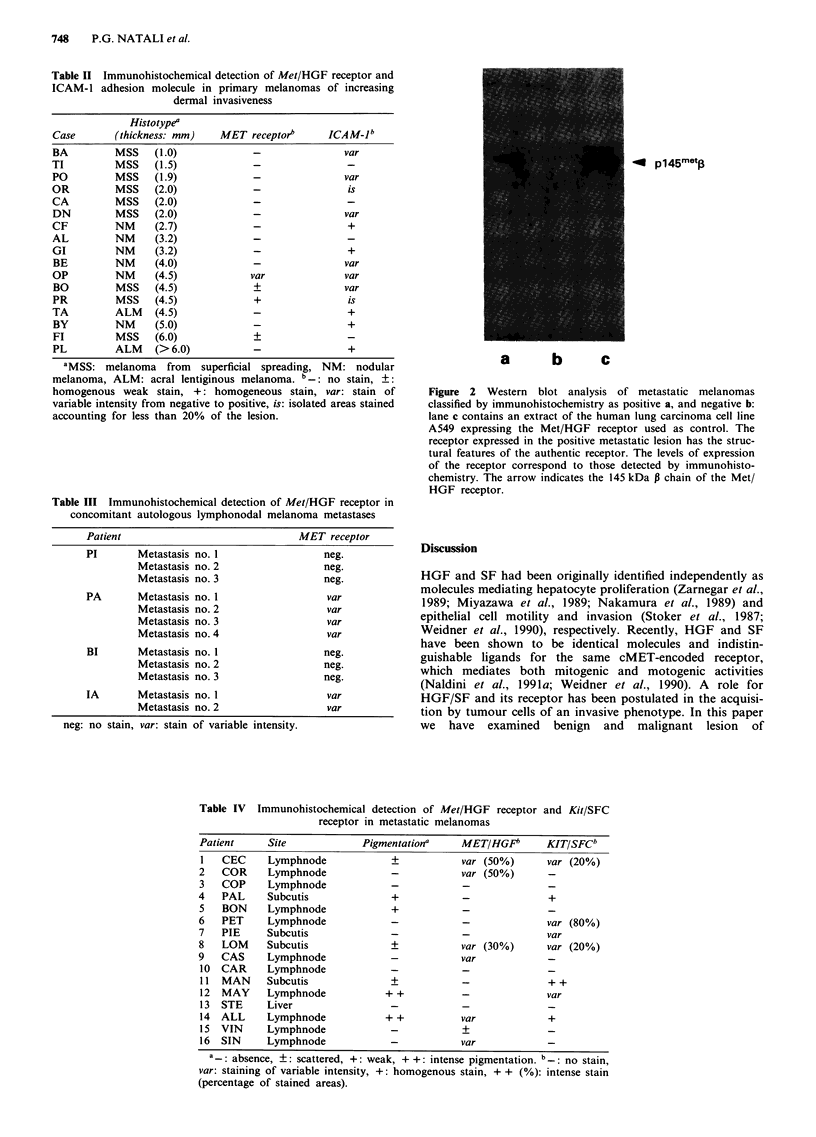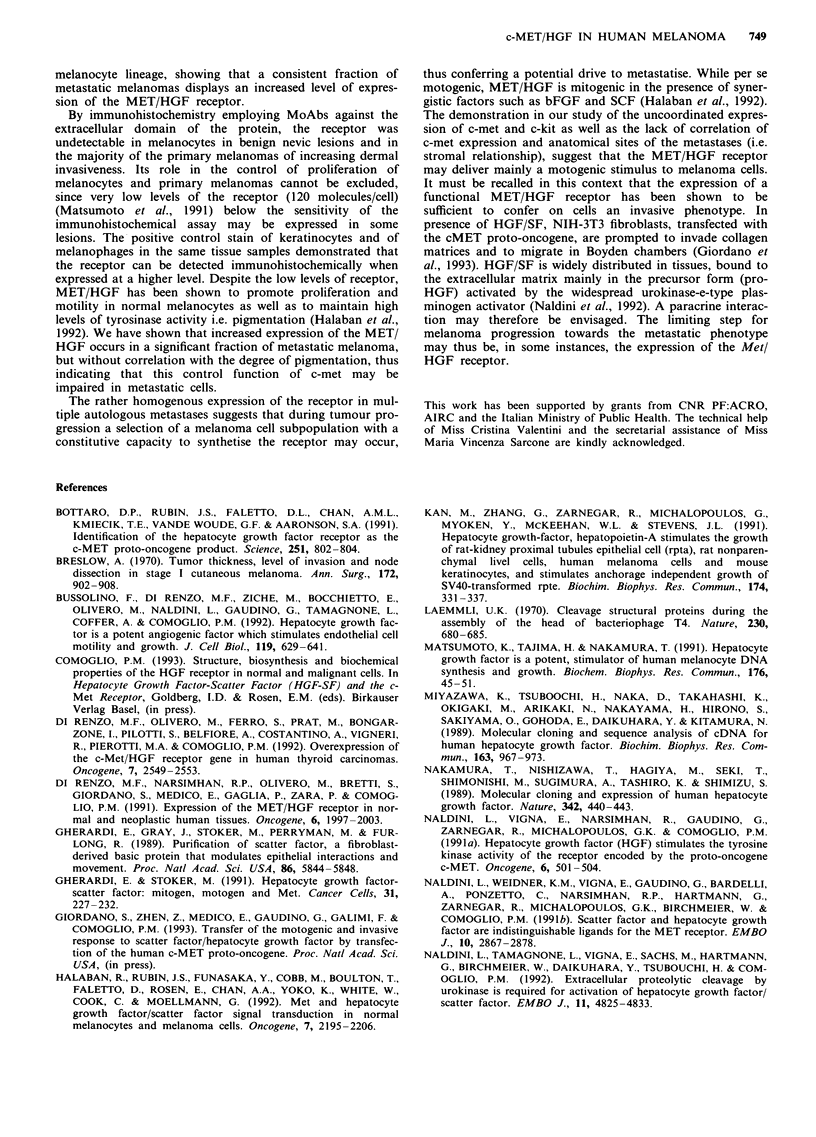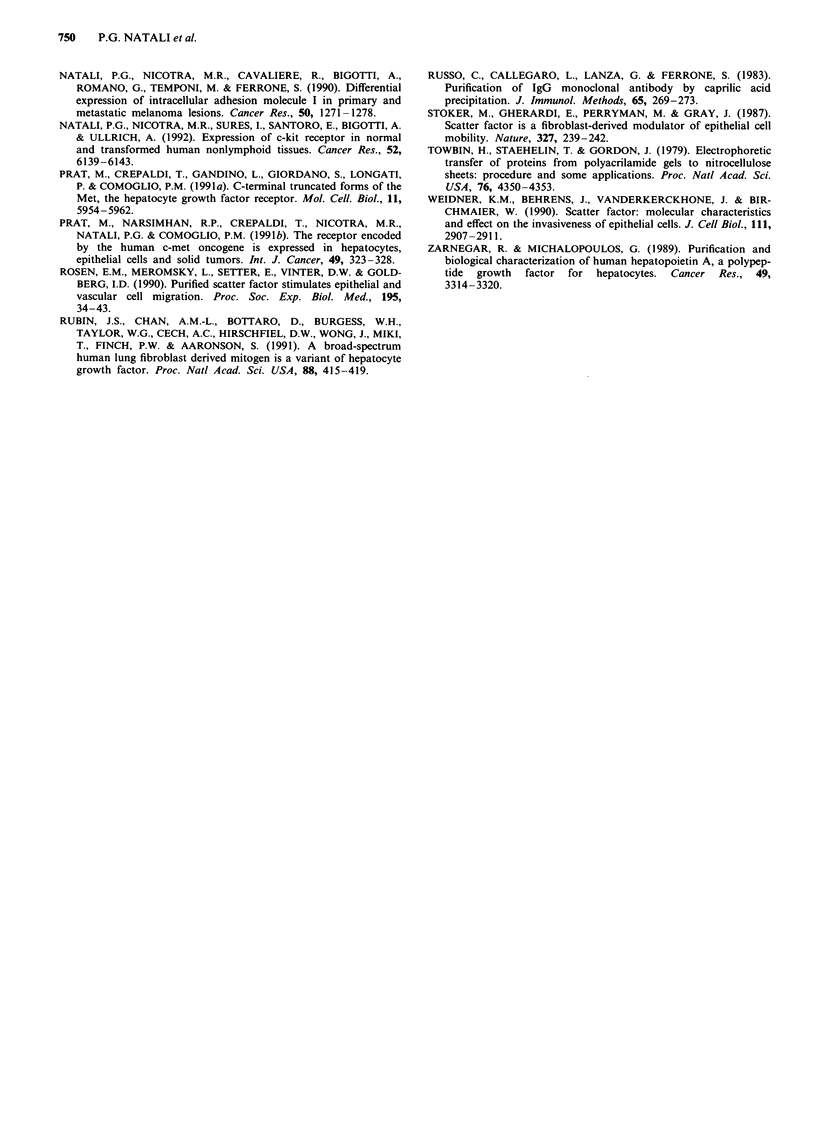# Expression of the c-Met/HGF receptor in human melanocytic neoplasms: demonstration of the relationship to malignant melanoma tumour progression.

**DOI:** 10.1038/bjc.1993.422

**Published:** 1993-10

**Authors:** P. G. Natali, M. R. Nicotra, M. F. Di Renzo, M. Prat, A. Bigotti, R. Cavaliere, P. M. Comoglio

**Affiliations:** Regina Elena Cancer Institute, Rome, Italy.

## Abstract

**Images:**


Br. J. Cancer (1993), 68, 746-750 © Macmillan Press Ltd., 1993

### Expression of the c-Met/HGF receptor in human melanocytic neoplasms: demonstration of the relationship to malignant melanoma tumour progression

P.G. Natali[1], M.R. Nicotra[2], M.F. Di Renzo[3], M. Prat[3], A. Bigotti[1], R. Cavaliere[1] & P.M. Comoglio[3]

[1]Regina Elena Cancer Institute, Rome; [2]Institute of Biomedical Technology CNR, Rome; [3]Department of Biomedical Sciences, University of Turin, Italy.

**Summary** The c-*MET* proto-oncogene encodes the receptor for the Hepatocyte Growth Factor/Scatter Factor, which is known to mediate mitogenic, motogenic and invasive responses of several cell types. We have analysed by immunohistochemistry and biochemically the expression of c-*MET* in benign and malignant melanocytic lesions. The *Met*/HGF receptor which in the melanocytic lineage displays the structural features of the authentic receptor was undetectable in tissue melanocytes and in nevocytic nevi. Only four out of 23 primary melanomas scored positive. Expression was increased to a significant level in 17 out of the 44 metastatic lesions examined. The c-*MET* expression was homogeneous in multiple metastases from the same patients. Comparative analyses showed both lack of correlation with the expression of the tumour progression associated ICAM-1 adhesion molecule and, in 23% of cases, co-expression with the c-*KIT* encoded receptor. These findings show that the c-*MET* gene is expressed at late stages of melanoma progression and suggest that the presence of *Met*/HGF receptor may contribute to the acquisition of an invasive phenotype.

The c-*MET* proto-oncogene encodes a transmembrane tyrosine kinase identified as the receptor for a polypeptide known as Hepatocyte Growth Factor (HGF) (Bottaro *et al.*, 1991; Naldini *et al.*, 1991*a*; Naldini *et al.*, 1991*b*). HGF (Miyazawa *et al.*, 1989; Nakamura *et al.*, 1989; Zarnegar *et al.*, 1989) is indistinguishable from Scatter Factor (SF), originally identified as a powerful polypeptide factor stimulating epithelial cell motility (Stoker *et al.*, 1987; Weidner *et al.*, 1990; Gherardi *et al.*, 1989). HGF/SF has been shown to exert a pleiotropic activity on several cell types mainly of epithelial origin. It is a powerful mitogen for hepatocytes both *in vitro* and *in vivo* (for a review see Gherardi and Stoker, 1991) and stimulates the growth *in vitro* of several other other epithelial cells, including kidney tubular cells, keratinocytes and endothelial cells (Kan *et al.*, 1991; Bussolino *et al.*, 1992; Rosen *et al.*, 1990; Rubin *et al.*, 1991). Interestingly, HGF/SF is a powerful inducer of epithelial cell dissociation, able to increase their motility and invasiveness (Weidner *et al.*, 1990). A role of HGF/SF and its receptor in promoting growth of tumour cells and/or in influencing their metastatic behaviour has been proposed (for a review see Comoglio, 1993). It has been recently shown that the *MET* encoded HGF/SF receptor is overexpressed in carcinomas of the GI tract (Di Renzo *et al.*, 1991) and in thyroid carcinomas belonging to clinically and histologically advanced subtypes (Di Renzo *et al.*, 1992). All this pointed to the involvement of HGF/SF and its receptor in the progression of tumour cells to a more malignant phenotype.

HGF/SF has been found to be mitogenic for melanocytes in primary cultures, mainly in the presence of synergistic factors (Matsumoto *et al.*, 1991; Rubin *et al.*, 1991; Halaban *et al.*, 1992) and its receptor has been detected in human melanocytes and melanomas grown *in vitro* (Kan *et al.*, 1991; Halaban *et al.*, 1992). In this paper, we studied the expression of the *Met*/HGF receptor in the natural history of human melanocytic lesions, by examining benign nevi, primary melanomas and metastases. We show that the expression of immunologically detectable *Met*/HGF receptor increases, with significant incidence (39%), in metastatic lesions, suggesting a correlation with the progression of melanoma.

Correspondence: P.G. Natali, Immunology Laboratory, Regina Elena Cancer Institute, Via delle Messi D'Oro, 156, 00158 Rome, Italy.
Received 11 March 1993; and in revised form 14 May 1993.

### Material and methods

### *Tissue specimens and cell lines*

Surgical biopsies of benign, malignant primary and metastatic melanocytic lesions were obtained from the Surgical Pathology section of the Regina Elena Cancer Institute. Tissue samples were snap frozen in liquid nitrogen. From each specimen 4 μm cryostat sections were obtained which were fixed in absolute acetone for 10 min. Fixed sections were either immediately used in immunohistochemical assays or kept frozen at − 70°C with no loss of serological activity. Fixed sections stained with 1% toluidine blue were used to evaluate the histological features of the lesions. Tumour thickness was evaluated according to Breslow (1970). Primary cultures of cutaneous melanocytes were obtained from Clonetics (San Diego, USA). Cytospins were prepared using a Shandon cytocentrifuge (Runcorn, Cheshire, UK).

### *Monoclonal antibodies*

The murine monoclonal antibody DQ13 against the α chain of *Met*/HGF receptor (Prat *et al.*, 1991*a*) was raised against a peptide corresponding to nineteen c-terminal amino acids (from $Ser^{1372}$ to $Ser^{1390}$) of the c-Met sequence, EMBL DataBank reference n X54559. The murine moAb DO24 to an epitope of the extracellular domain of the c-met gene product was produced using the human gastric carcinoma cell line GTL-16 as immunogen (Prat *et al.*, 1991*b*). MoAb 84h10 to the intercellular adhesion molecule ICAM-1 was obtained from Immunotech, Marseille, France. MoAb to the extracellular domain of the c-kit receptor was purchased from Boehringer Mannheim.

### *Serological assays*

The immunohistochemical analysis employed MoAb DO24 as purified reagents (Russo *et al.*, 1983) at concentrations ranging from 10 to 50 $\mu g\, ml^{-1}$. The indirect immunoperoxidase stain was performed with commercially available reagents (Vectastain Elite, Burlingame, Ca. USA). Slides were incubated overnight with MoAb at 4°C in a moist chamber. The enzymatic activity was developed using 3-amino-9-ethylcarbazole (AEC) as chromogenic substrate for 8 min. Slides were then rinsed with phosphate buffered saline and counterstained with Mayer's hematoxylin. Sections on which the incubation of the primary antibody was omitted were used as control.

### Western Blotting

Surgical biopsies immediately frozen in liquid nitrogen were pulverised using a Mikro-Dismembrator TM (B-Braun) in the presence of liquid nitrogen. Powdered tissues were solubilised in boiling Laemmli buffer (Laemmli, 1970), containing the reducing agent β-mercaptoethanol. Four hundred μg of proteins were loaded on each lane. Western blot analysis was carried out as described by Towbin *et al.* (1979). Bound antibodies were revealed with rabbit anti-mouse antibodies labelled with HR Peroxidase according to the enhanced chemiluminescence method (ECL™, Amersham).

### Results

Table I shows the list of melanocytic samples examined by immunohistochemistry. Cultured melanocytes, nevus cells of intra-dermal and junctional nevi were not labelled by the monoclonal antibody directed against the extracellular domain of the MET/HGF receptor (Figure 1a). In the same biopsies MoAb DO24 stained with variable intensity the keratinocytes of the basal layer as well as melanophages. Similarly the receptor was not histochemically detectable in the majority of primary tumours (Table I). However four lesions were scored positive, showing either a homogenous-weak staining or an irregular distribution of the antigen. The four positive tumours displayed a high degree of dermal invasiveness (Figure 1b). For comparison the pattern of expression in the same lesions of the ICAM-1 molecule which is known to be associated with increasing tumour invasiveness (Natali *et al.*, 1990) is reported (Table II). Positive staining with the ICAM-1 monoclonal antibody was observed with the expected distribution without a significant correlation with the expression of the *MET*/HGF receptor. In contrast to primary tumours 17 out of 44 (39%) metastatic foci collected from different body sites, showed a positive staining with an apparent lower incidence in parenchymal metastases. The intensity of the stain was generally weak and only in a minority of the instances was homogenously distributed in the metastatic cell population (Figure 1c). Evaluation in four patients of individual concomitant metastases (Table III) showed that the expression of the gene product is rather consistent among autologous lesions. Because the expression of the c-kit receptor for Stem Cell Factor (SCF) has been shown to be downregulated in human melanocytes following malignant transformation (Natali *et al.*, 1992) a comparative analysis of the c-met and c-kit products in 16 unselected metastatic lesions was performed. The results reported on Table IV demonstrated that the two receptor are uncoordinately expressed with only 20% of the samples displaying detectable levels of both molecules. Furthermore no relationship was found between expression of c-met and degree of pigmentation. In order to investigate the molecular structure of the c-met receptor expressed by melanoma cells, selected biopsies expressing different levels of c-met were also analysed by Western blotting employing MoAb DQ13 against the COOH-terminal tail of the human protein. Figure 2 shows that the *MET*/HGF receptor of melanocytic lesions has structural features of the authentic receptor. The levels of expression of the MET/HGF receptor detected in Western blot analysis corresponded to those detected by immunohistochemistry.

**Table I** Immunohistochemical detection of *Met*/HGF receptor in benign and malignant lesions of the melanocyte lineage

| | |
|---|---|
| Melanocytes (primary cultures) | neg. |
| Intradermal-junctional nevi | 0/15[a] |
| Blue nevi (simple and cellular type) | 0/5 |
| Primary melanomas: | |
| melanoma *in situ* | 0/3 |
| melanoma from superficial spreading | 3/12 |
| nodular melanoma | 1/6 |
| acral lentiginous melanoma | 0/2 |
| total | 4/23 |
| Metastatic melanomas: | |
| lymphonodal | 13/33 |
| cutaneous | 3/7 |
| parenchymal | 1/4 |
| total | 17/44 |

[a]No. positive/no. tested.

**Figure 1** Expression of Met/HGF receptor in melanocyte lesions as revealed by indirect avidin/biotin immunoperoxidase employing MoAb DO24 on 4 μ acetone fixed cryostat sections. No detectable levels of the receptor are expressed by nevus cells of an intra-dermal nevus (asterisk). A weak stain is present in basal keratinocytes (arrow) **a**. Melanoma cells and melanophages (arrows) of a primary melanoma display variable levels of immunoreactivity **b**. The immunoreactivity of the latter cells is partially shadowed by the melanin pigment. The receptor is homogenously expressed in a case of metastatic melanoma **c**. (**a**–**c**: × 200).

**Table II** Immunohistochemical detection of *Met*/HGF receptor and ICAM-1 adhesion molecule in primary melanomas of increasing dermal invasiveness

| Case | Histotype[a] (thickness: mm) | | MET receptor[b] | ICAM-1[b] |
|---|---|---|---|---|
| BA | MSS | (1.0) | – | *var* |
| TI | MSS | (1.5) | – | – |
| PO | MSS | (1.9) | – | *var* |
| OR | MSS | (2.0) | – | *is* |
| CA | MSS | (2.0) | – | – |
| DN | MSS | (2.0) | – | *var* |
| CF | NM | (2.7) | – | + |
| AL | NM | (3.2) | – | – |
| GI | NM | (3.2) | – | + |
| BE | NM | (4.0) | – | *var* |
| OP | NM | (4.5) | *var* | *var* |
| BO | MSS | (4.5) | ± | *var* |
| PR | MSS | (4.5) | + | *is* |
| TA | ALM | (4.5) | – | + |
| BY | NM | (5.0) | – | + |
| FI | MSS | (6.0) | ± | – |
| PL | ALM | (>6.0) | – | + |

[a]MSS: melanoma from superficial spreading, NM: nodular melanoma, ALM: acral lentiginous melanoma. [b]–: no stain, ±: homogenous weak stain, +: homogeneous stain, *var*: stain of variable intensity from negative to positive, *is*: isolated areas stained accounting for less than 20% of the lesion.

**Table III** Immunohistochemical detection of *Met*/HGF receptor in concomitant autologous lymphonodal melanoma metastases

| Patient | | MET receptor |
|---|---|---|
| PI | Metastasis no. 1 | neg. |
| | Metastasis no. 2 | neg. |
| | Metastasis no. 3 | neg. |
| PA | Metastasis no. 1 | *var* |
| | Metastasis no. 2 | *var* |
| | Metastasis no. 3 | *var* |
| | Metastasis no. 4 | *var* |
| BI | Metastasis no. 1 | neg. |
| | Metastasis no. 2 | neg. |
| | Metastasis no. 3 | neg. |
| IA | Metastasis no. 1 | *var* |
| | Metastasis no. 2 | *var* |

neg: no stain, *var*: stain of variable intensity.

**Figure 2** Western blot analysis of metastatic melanomas classified by immunohistochemistry as positive **a**, and negative **b**: lane **c** contains an extract of the human lung carcinoma cell line A549 expressing the Met/HGF receptor used as control. The receptor expressed in the positive metastatic lesion has the structural features of the authentic receptor. The levels of expression of the receptor correspond to those detected by immunohistochemistry. The arrow indicates the 145 kDa $\beta$ chain of the Met/HGF receptor.

### Discussion

HGF and SF had been originally identified independently as molecules mediating hepatocyte proliferation (Zarnegar *et al.*, 1989; Miyazawa *et al.*, 1989; Nakamura *et al.*, 1989) and epithelial cell motility and invasion (Stoker *et al.*, 1987; Weidner *et al.*, 1990), respectively. Recently, HGF and SF have been shown to be identical molecules and indistinguishable ligands for the same cMET-encoded receptor, which mediates both mitogenic and motogenic activities (Naldini *et al.*, 1991*a*; Weidner *et al.*, 1990). A role for HGF/SF and its receptor has been postulated in the acquisition by tumour cells of an invasive phenotype. In this paper we have examined benign and malignant lesion of

**Table IV** Immunohistochemical detection of *Met*/HGF receptor and *Kit*/SFC receptor in metastatic melanomas

| Patient | | Site | Pigmentation[a] | MET/HGF[b] | KIT/SFC[b] |
|---|---|---|---|---|---|
| 1 | CEC | Lymphnode | ± | *var* (50%) | *var* (20%) |
| 2 | COR | Lymphnode | – | *var* (50%) | – |
| 3 | COP | Lymphnode | – | – | – |
| 4 | PAL | Subcutis | + | – | + |
| 5 | BON | Lymphnode | + | – | – |
| 6 | PET | Lymphnode | – | – | *var* (80%) |
| 7 | PIE | Subcutis | – | – | *var* |
| 8 | LOM | Subcutis | ± | *var* (30%) | *var* (20%) |
| 9 | CAS | Lymphnode | – | *var* | – |
| 10 | CAR | Lymphnode | – | – | – |
| 11 | MAN | Subcutis | ± | – | + + |
| 12 | MAY | Lymphnode | + + | – | *var* |
| 13 | STE | Liver | – | – | – |
| 14 | ALL | Lymphnode | + + | *var* | + |
| 15 | VIN | Lymphnode | – | ± | – |
| 16 | SIN | Lymphnode | – | *var* | – |

[a]–: absence, ±: scattered, +: weak, + +: intense pigmentation. [b]–: no stain, *var*: staining of variable intensity, +: homogenous stain, + + (%): intense stain (percentage of stained areas).

melanocyte lineage, showing that a consistent fraction of metastatic melanomas displays an increased level of expression of the MET/HGF receptor.

By immunohistochemistry employing MoAbs against the extracellular domain of the protein, the receptor was undetectable in melanocytes in benign nevic lesions and in the majority of the primary melanomas of increasing dermal invasiveness. Its role in the control of proliferation of melanocytes and primary melanomas cannot be excluded, since very low levels of the receptor (120 molecules/cell) (Matsumoto *et al.*, 1991) below the sensitivity of the immunohistochemical assay may be expressed in some lesions. The positive control stain of keratinocytes and of melanophages in the same tissue samples demonstrated that the receptor can be detected immunohistochemically when expressed at a higher level. Despite the low levels of receptor, MET/HGF has been shown to promote proliferation and motility in normal melanocytes as well as to maintain high levels of tyrosinase activity i.e. pigmentation (Halaban *et al.*, 1992). We have shown that increased expression of the MET/HGF occurs in a significant fraction of metastatic melanoma, but without correlation with the degree of pigmentation, thus indicating that this control function of c-met may be impaired in metastatic cells.

The rather homogenous expression of the receptor in multiple autologous metastases suggests that during tumour progression a selection of a melanoma cell subpopulation with a constitutive capacity to synthetise the receptor may occur, thus conferring a potential drive to metastatise. While per se motogenic, MET/HGF is mitogenic in the presence of synergistic factors such as bFGF and SCF (Halaban *et al.*, 1992). The demonstration in our study of the uncoordinated expression of c-met and c-kit as well as the lack of correlation of c-met expression and anatomical sites of the metastases (i.e. stromal relationship), suggest that the MET/HGF receptor may deliver mainly a motogenic stimulus to melanoma cells. It must be recalled in this context that the expression of a functional MET/HGF receptor has been shown to be sufficient to confer on cells an invasive phenotype. In presence of HGF/SF, NIH-3T3 fibroblasts, transfected with the cMET proto-oncogene, are prompted to invade collagen matrices and to migrate in Boyden chambers (Giordano *et al.*, 1993). HGF/SF is widely distributed in tissues, bound to the extracellular matrix mainly in the precursor form (pro-HGF) activated by the widespread urokinase-e-type plasminogen activator (Naldini *et al.*, 1992). A paracrine interaction may therefore be envisaged. The limiting step for melanoma progression towards the metastatic phenotype may thus be, in some instances, the expression of the *Met*/HGF receptor.

This work has been supported by grants from CNR PF:ACRO, AIRC and the Italian Ministry of Public Health. The technical help of Miss Cristina Valentini and the secretarial assistance of Miss Maria Vincenza Sarcone are kindly acknowledged.

### References

BOTTARO, D.P., RUBIN, J.S., FALETTO, D.L., CHAN, A.M.L., KMIECIK, T.E., VANDE WOUDE, G.F. & AARONSON, S.A. (1991). Identification of the hepatocyte growth factor receptor as the c-MET proto-oncogene product. *Science*, **251**, 802–804.

BRESLOW, A. (1970). Tumor thickness, level of invasion and node dissection in stage I cutaneous melanoma. *Ann. Surg.*, **172**, 902–908.

BUSSOLINO, F., DI RENZO, M.F., ZICHE, M., BOCCHIETTO, E., OLIVERO, M., NALDINI, L., GAUDINO, G., TAMAGNONE, L., COFFER, A. & COMOGLIO, P.M. (1992). Hepatocyte growth factor is a potent angiogenic factor which stimulates endothelial cell motility and growth. *J. Cell Biol.*, **119**, 629–641.

COMOGLIO, P.M. (1993). Structure, biosynthesis and biochemical properties of the HGF receptor in normal and malignant cells. In *Hepatocyte Growth Factor-Scatter Factor (HGF-SF) and the c-*Met *Receptor*, Goldberg, I.D. & Rosen, E.M. (eds). Birkauser Verlag Basel, (in press).

DI RENZO, M.F., OLIVERO, M., FERRO, S., PRAT, M., BONGARZONE, I., PILOTTI, S., BELFIORE, A., COSTANTINO, A., VIGNERI, R., PIEROTTI, M.A. & COMOGLIO, P.M. (1992). Overexpression of the c-Met/HGF receptor gene in human thyroid carcinomas. *Oncogene*, **7**, 2549–2553.

DI RENZO, M.F., NARSIMHAN, R.P., OLIVERO, M., BRETTI, S., GIORDANO, S., MEDICO, E., GAGLIA, P., ZARA, P. & COMOGLIO, P.M. (1991). Expression of the MET/HGF receptor in normal and neoplastic human tissues. *Oncogene*, **6**, 1997–2003.

GHERARDI, E., GRAY, J., STOKER, M., PERRYMAN, M. & FURLONG, R. (1989). Purification of scatter factor, a fibroblast-derived basic protein that modulates epithelial interactions and movement. *Proc. Natl Acad. Sci. USA*, **86**, 5844–5848.

GHERARDI, E. & STOKER, M. (1991). Hepatocyte growth factor-scatter factor: mitogen, motogen and Met. *Cancer Cells*, **31**, 227–232.

GIORDANO, S., ZHEN, Z., MEDICO, E., GAUDINO, G., GALIMI, F. & COMOGLIO, P.M. (1993). Transfer of the motogenic and invasive response to scatter factor/hepatocyte growth factor by transfection of the human c-MET proto-oncogene. *Proc. Natl Acad. Sci. USA*, (in press).

HALABAN, R., RUBIN, J.S., FUNASAKA, Y., COBB, M., BOULTON, T., FALETTO, D., ROSEN, E., CHAN, A.A., YOKO, K., WHITE, W., COOK, C. & MOELLMANN, G. (1992). Met and hepatocyte growth factor/scatter factor signal transduction in normal melanocytes and melanoma cells. *Oncogene*, **7**, 2195–2206.

KAN, M., ZHANG, G., ZARNEGAR, R., MICHALOPOULOS, G., MYOKEN, Y., MCKEEHAN, W.L. & STEVENS, J.L. (1991). Hepatocyte growth-factor, hepatopoietin-A stimulates the growth of rat-kidney proximal tubules epithelial cell (rpta), rat nonparenchymal livel cells, human melanoma cells and mouse keratinocytes, and stimulates anchorage independent growth of SV40-transformed rpte. *Biochim. Biophys. Res. Commun.*, **174**, 331–337.

LAEMMLI, U.K. (1970). Cleavage structural proteins during the assembly of the head of bacteriophage T4. *Nature*, **230**, 680–685.

MATSUMOTO, K., TAJIMA, H. & NAKAMURA, T. (1991). Hepatocyte growth factor is a potent, stimulator of human melanocyte DNA synthesis and growth. *Biochem. Biophys. Res. Commun.*, **176**, 45–51.

MIYAZAWA, K., TSUBOOCHI, H., NAKA, D., TAKAHASHI, K., OKIGAKI, M., ARIKAKI, N., NAKAYAMA, H., HIRONO, S., SAKIYAMA, O., GOHODA, E., DAIKUHARA, Y. & KITAMURA, N. (1989). Molecular cloning and sequence analysis of cDNA for human hepatocyte growth factor. *Biochim. Biophys. Res. Commun.*, **163**, 967–973.

NAKAMURA, T., NISHIZAWA, T., HAGIYA, M., SEKI, T., SHIMONISHI, M., SUGIMURA, A., TASHIRO, K. & SHIMIZU, S. (1989). Molecular cloning and expression of human hepatocyte growth factor. *Nature*, **342**, 440–443.

NALDINI, L., VIGNA, E., NARSIMHAN, R., GAUDINO, G., ZARNEGAR, R., MICHALOPOULOS, G.K. & COMOGLIO, P.M. (1991*a*). Hepatocyte growth factor (HGF) stimulates the tyrosine kinase activity of the receptor encoded by the proto-oncogene c-MET. *Oncogene*, **6**, 501–504.

NALDINI, L., WEIDNER, K.M., VIGNA, E., GAUDINO, G., BARDELLI, A., PONZETTO, C., NARSIMHAN, R.P., HARTMANN, G., ZARNEGAR, R., MICHALOPOULOS, G.K., BIRCHMEIER, W. & COMOGLIO, P.M. (1991*b*). Scatter factor and hepatocyte growth factor are indistinguishable ligands for the MET receptor. *EMBO J.*, **10**, 2867–2878.

NALDINI, L., TAMAGNONE, L., VIGNA, E., SACHS, M., HARTMANN, G., BIRCHMEIER, W., DAIKUHARA, Y., TSUBOUCHI, H. & COMOGLIO, P.M. (1992). Extracellular proteolytic cleavage by urokinase is required for activation of hepatocyte growth factor/scatter factor. *EMBO J.*, **11**, 4825–4833.

NATALI, P.G., NICOTRA, M.R., CAVALIERE, R., BIGOTTI, A., ROMANO, G., TEMPONI, M. & FERRONE, S. (1990). Differential expression of intracellular adhesion molecule I in primary and metastatic melanoma lesions. *Cancer Res.*, **50**, 1271–1278.

NATALI, P.G., NICOTRA, M.R., SURES, I., SANTORO, E., BIGOTTI, A. & ULLRICH, A. (1992). Expression of c-kit receptor in normal and transformed human nonlymphoid tissues. *Cancer Res.*, **52**, 6139–6143.

PRAT, M., CREPALDI, T., GANDINO, L., GIORDANO, S., LONGATI, P. & COMOGLIO, P.M. (1991*a*). C-terminal truncated forms of the Met, the hepatocyte growth factor receptor. *Mol. Cell. Biol.*, **11**, 5954–5962.

PRAT, M., NARSIMHAN, R.P., CREPALDI, T., NICOTRA, M.R., NATALI, P.G. & COMOGLIO, P.M. (1991*b*). The receptor encoded by the human c-met oncogene is expressed in hepatocytes, epithelial cells and solid tumors. *Int. J. Cancer*, **49**, 323–328.

ROSEN, E.M., MEROMSKY, L., SETTER, E., VINTER, D.W. & GOLDBERG, I.D. (1990). Purified scatter factor stimulates epithelial and vascular cell migration. *Proc. Soc. Exp. Biol. Med.*, **195**, 34–43.

RUBIN, J.S., CHAN, A.M.-L., BOTTARO, D., BURGESS, W.H., TAYLOR, W.G., CECH, A.C., HIRSCHFIEL, D.W., WONG, J., MIKI, T., FINCH, P.W. & AARONSON, S. (1991). A broad-spectrum human lung fibroblast derived mitogen is a variant of hepatocyte growth factor. *Proc. Natl Acad. Sci. USA*, **88**, 415–419.

RUSSO, C., CALLEGARO, L., LANZA, G. & FERRONE, S. (1983). Purification of IgG monoclonal antibody by caprilic acid precipitation. *J. Immunol. Methods*, **65**, 269–273.

STOKER, M., GHERARDI, E., PERRYMAN, M. & GRAY, J. (1987). Scatter factor is a fibroblast-derived modulator of epithelial cell mobility. *Nature*, **327**, 239–242.

TOWBIN, H., STAEHELIN, T. & GORDON, J. (1979). Electrophoretic transfer of proteins from polyacrilamide gels to nitrocellulose sheets: procedure and some applications. *Proc. Natl Acad. Sci. USA*, **76**, 4350–4353.

WEIDNER, K.M., BEHRENS, J., VANDERKERCKHONE, J. & BIRCHMAIER, W. (1990). Scatter factor: molecular characteristics and effect on the invasiveness of epithelial cells. *J. Cell Biol.*, **111**, 2907–2911.

ZARNEGAR, R. & MICHALOPOULOS, G. (1989). Purification and biological characterization of human hepatopoietin A, a polypeptide growth factor for hepatocytes. *Cancer Res.*, **49**, 3314–3320.